# Report of Meeting of Obstetrical Society of Cincinnati

**Published:** 1893-08

**Authors:** 


### REPORT OF MEETING OF OBSTETRICAL SOCIETY OF CINCINNATI.

---

The role of the pessary in the cure of retro-displacements of the uterus was the subject of a paper read before the Obstetrical Society of Cincinnati, by Dr. Sigmar Stark. In this paper the doctor made the claim that backward displacements of uterus were cured by the pessary through the induction of an inflammatory state in these ligaments.

Dr. Palmer. Mr. President: We all must realize that this is an important subject. It requires a very good judgment to know when to use, and when not to use, a pessary. I believe that pessaries are out of fashion now-a-days, but when I commenced practice, pessaries were in fashion. I think I used a pessary twenty-five times more frequently fifteen years ago than I do to-day. Nevertheless, I am by no means prejudiced against the nse of them, but I believe that now pessaries can largely be dispensed with. I don't believe that we need them nearly so frequently as we formerly did. Displacement of the uterus, whether downward, forward or even upward, is not really a disease, but only a secondary condition. I think Dr. Thomas was the first man who enunciated this principle of practice, and with his usual clearness and force. He stated that all displacements are dependent upon four causes: first, increased bulk and weight, causing the uterus to descend, or become displaced forward or backward; second, relaxation of support; third, increased pressure from above, and fourth, increased traction from below. Always it will depend upon one or more of these factors. No one would think of attempting to treat a uterus displaced because of relaxation in support, or increased weight from above, or increased traction from below, by the methods useful for the relief of displacement caused by increased bulk and weight. We should rectify, as far as we can, any of the ætiological factors, and having done that, I am disposed to think that, most displacements of the uterus, including posterior displacements, of course, will be relieved of their morbid symptoms. In almost all displacements of the uterus, there is an error in the circulation, as well as an error in place, and it is this error in circulation that creates the symptoms as well as the error in place. How many times have we seen this morbid condition, and how often we have seen complete displacement without any complaint whatsoever. I do not know but that retroversion is just as common as prolapsus, for we never have retroversion without prolapsus, as we never have prolapsus without retroversion. Mechanically they go together. A pessary should not be used, until the anterior conditions creating or co-existing have been corrected, as far as practicable. If there are adhesions, of course they are to be modified or removed.

There is another method of treatment, of which I desire to speak, and that is the use of the Faradic current. I think the Faradic current is a potent factor in the treatment of some of these cases of displacement of the uterus. Remove all anterior conditions, as far as practicable, and then replace the uterus, aided it may be by a proper electrode, and with the latter in position, retaining the uterus in situ, we utilize the primary Faradic current, with the negative pole placed in the posterior vaginal cul-de-sac,—unless there are menstrual objections—and the other pole on the abdomen. Thus the current will pass through the sacro-uterine ligaments, as well as through the recto-uterine ligaments, and through the uterus itself. I believe we seldom have these conditions unless there is some pain felt in the uterus, and generally there is a bulk in weight, as well as a relaxation of support. Generally pessaries relieve for the time being, and as soon as withdrawn, the uterus falls back again into its faulty posture. Then they become palliative only, and not curative. Exceptionally, however, they are curative.

Management of the Third Stage of Labor, was the subject of a paper before the Cincinnati Obstetrical Society, by W. D. Porter, A.M., M. D.

The doctor said that no difference what was the idea of the physician on the subject of antiseptics he should make his examinations during the third stage with clean hands. Great stress was laid on susceptibility of women during labor. An examination with an unclean hand under such conditions results in literally grinding infectious material into the gaping arteries, veins and lymphatics. Efforts at expulsion of the placenta previous to its detachment are clearly wrong. The doctors method of delivering the placenta is as follows : Soon after the birth of the child the uterus is grasped with both hands but no compression is made until one or two pains have occurred. Then with each pain firm compression is made, but as a rule not so forcibly as to cause the patient to protest, nor is the uterus crowded into the pelvis. In nearly every instance the placenta is felt to slip down from its attachment. Then without attempting to carry the process to a point of complete expulsion an aseptic finger is carried into the vagina, hooked into the lower portion of the placenta and gentle traction made sufficient to overcome the friction of the forward part against the vaginal walls and to prevent the thickening and folding of the mass from the vis-a-tergo. At the same time the other hand makes downward pressure over the fundus in conjunction with uterine contraction. After the placenta is out of the uterus it is moved forward gently and if the membranes do not readily follow they may be extracted during relaxation of the uterus.

Rigidity of The Os Uteri was the subject of a paper before the Obstetrical Society of Cincinnati, by Dr. E. W. Mitchell. He considered the finger useful at times, but thought extreme care necessary that this means does not produce or if present increase spasm of the muscular fibres. The colpeurynter is much more efficient and safe where mechanical dilatation is called for. Electricity deserved much more attention as a powerful uterine stimulant than it received. He has found quinine to increase the uterine contraction notably. He finds chloroform advantageous even in the first stage where it is generally considered inadmisable. When other measures fail incisions in the rigid os may be made. They should be made under antiseptic precautions and need not be deep, one-fourth to one-sixth inch.

Inter-ligamentary Cyst was the subject of a report by Dr. T. A. Ramy before the Obstetrical Society of Cincinnati. This tumor, which weighed 45 pounds, was removed from a woman aged 70, at the Good Samaritan Hospital. It was an inter-ligamentary cyst, and the blood supply was obtained from the omentum through vessels half as large as a finger. I was able to ligate it from low down, in the ordinary way. I had stopped all bleeding points and was closing the wound up without drainage. When I was about to close the lower stitch, I carried a sponge down, as is my custom in such cases, and it came up filled with blood. I repeated this and it was again filled with blood. On examination I found nearly a pint of blood in the pelvis. I was satisfied this could not be due to oozing and cutting two stitches I could easily see the blood was coming from the pedicle. But when I brought the pedicle up I found it secure. I had taken the precaution to treat separately the ovarian artery and another large artery, and I could see the hemorrhage was coming from the side of the pedicle. On turning it over I found a very large vein had been ruptured below my ligature, and the folds of the pedicle kept it from bleeding until the fold was removed by stitching The vein was as large as a goose quill and the hemorrhage would probably have caused the death of the patient had it not been found. I think this justifies the practice of carrying down a sponge before closing the wound to find if there is any hemorrhage present.

